# Regularities of Anthocyanins Retention in RP HPLC for “Water–Acetonitrile–Phosphoric Acid” Mobile Phases

**DOI:** 10.1155/2015/732918

**Published:** 2015-01-26

**Authors:** V. I. Deineka, L. A. Deineka, I. I. Saenko

**Affiliations:** Faculty of Biology and Chemistry, Federal State Autonomous Educational Institution of Higher Professional Education “Belgorod National Research University”, Pobeda Street 85, Belgorod 308015, Russia

## Abstract

The influence of exchange of HCOOH (System 2) by phosphoric acid (System 1) for acidification of the “acetonitrile–water” mobile phases for reversed-phase HPLC of anthocyanins was investigated in the framework of relative retention analysis. The differences and similarities of anthocyanins separation were revealed. It has been shown that some common features of the quantitative relationships may be used for preliminary anthocyanins structure differentiation, according to the number of OH-groups in anthocyanidin backbone as well as to a number of saccharide molecules in glycoside radicals in position 3 of the anthocyanin without MS detection.

## 1. Introduction

**Figure 1:**
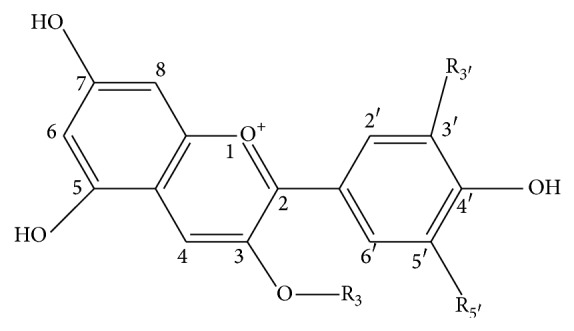
Flavylium form of 3-glycosides of six common anthocyanidins. R_3′_ and R_5′_ are H and H for pelargonidin (Pg); OH and H for cyanidin (Cy); OH and OH for delphinidin (Dp); OCH_3_ and H for peonidin (Pn); OCH_3_ and OH for petunidin (Pt); OCH_3_ and OCH_3_ for malvidin (Mv).

Anthocyanins are powerful water-soluble antioxidants of flavonoids class with health promoting effect [1, 2]. The coloured flavylium form of anthocyanins is a reason to regard them as natural food colorants [2]. The latter explains a high scientific and technological interest to the substances. Anthocyanins are synthesized in plant tissue, mainly in fruits, flowers and for some species in leaves as a rule as a complex mixture of compounds with different structures [3]. Anthocyanins are glycosides of anthocyanidins (Figure 1), with great varieties of more than 600 anthocyanin structures found in plant sources [4] though only six structures of the latter cover the majority of the structures due to glycosylation type variability [5].

Reversed-phase HPLC is a common method for analysis of complex mixtures of plant anthocyanins [6, 7]. The specificity of the method is usage of rather strong acidic mixtures of water and organic water-miscible solvent. Acidification is necessary to transfer the substances into charged and coloured flavylium form, due to the fact that anthocyanins may be easily detected at the presence of large amounts of other colourless substances.

The retention of substances in HPLC depends upon type and even trademark of stationary reversed phase, composition of mobile phase, and temperature as well as solute structure. Mobile phases of water mixtures with acetonitrile or methanol are acidified with HCOOH [8], acetic [9], phosphoric [10], and trifluoroacetic [11] acids as well as the mixtures (without any explanation) of some of them [12–14] for anthocyanins separation. However, as far as we know only “HCOOH–acetonitrile–water” mobile phases were investigated extensively to elucidate the regularities of anthocyanins retention [8, 15–17]. According to Snyder' selectivity triangular water, acetonitrile and HCOOH are solvents of different groups, VIII, VIb, and IV correspondingly [18], so the withdrawal or exchange of some solvents may lead to alteration of solutes separation selectivity.

The aim of the present paper is the investigation of regularities of anthocyanins retention in RP HPLC with mobile phases composed of water, acetonitrile and phosphoric acid and comparing that with retention in the more commonly used mobile phases being water–acetonitrile mixtures acidified with HCOOH (10 vol. %).

## 2. Experimental

### 2.1. Chemicals and Reagents

All anthocyanins under investigation were extracted from the plant sources and isolated by means of semipreparative HPLC. 3-Glucosides of delphinidin (Dp3Glu), cyanidin (Cy3Glu), petunidin (Pt3Glu), peonidin (Pn3Glu), and malvidin (Mv3Glu) were isolated from grape fruit skin; pelargonidin-3-glucoside (Pg3Glu) was isolated from strawberry fruits; cyanidin-3-sophoroside (Cy3Sopho) and cyanidin-3-glucosylrutinoside (Cy3GRut) were isolated from sour cherry fruits; cyanidin-3-sambubioside (Cy3Sam) and cyanidin-3-xylosilrutinosyde (Cy3XRut) were isolated from red currant fruits; cyanidin-3-arabinosylglucoside (Cy3AGlu) was isolated from rowanberry fruits; cyanidin-3-galactoside (Cy3Gala) and cyanidin-3-arabinoside (Cy3Ara) were isolated from saskatoon fruit. The solute structures were confirmed by electronic and mass spectrum with relative retention control [16, 17].

Mobile phases for HPLC were composed of distilled water, acetonitrile (Super Gradient, LAB-SCAN), reagent grade HCOOH, and phosphoric acid (SPECTR-CHEM Ltd., RF).

### 2.2. Extraction and Partial Purification of Anthocyanins

Plant material was dispersed in 0.1 M solution of HCl in distilled water with blander and macerated overnight. Then supernatant was decanted and applied for solid phase extraction by DIAPAC C18 cartridges (BioChemMak ST, RF).

### 2.3. Semipreparative Isolation of Individual Anthocyanins

The isolation of individual anthocyanins was performed by Shimadzu equipment LC-20 with spectrophotometric detection on chromatographic column 10 × 250 mm SUPELCOSIL C18 (5 mcm) in eluents of water–acetonitrile, 10 vol. % HCOOH system.

### 2.4. Analytical HPLC

The fractions of semipreparative separations were controlled and chromatographic behaviour of anthocyanins was investigated with utilisation of Agilent Infinity 1200 equipment with diode array (DAD) and MS (6130 Quadrupole LC/MS) detectors. Chromatographic column: 4.6 × 250 mm Symmetry C_18_ in two mobile phase systems.

*System  1*: solutions of acetonitrile in distilled water (8–14 vol. %), acidified with phosphoric acid (0.5 vol. %). The concentration of phosphoric acid (0.5 vol. %) in System 1 was chosen to match the pH close to that for 10 vol. % of HCOOH (1.45–1.50) of System 2. It should be mentioned that the decrease of acid content leads to increase of mobile phase pH and to decrease of number theoretical plates of the chromatographic system under investigation [19].

Reference* System  2*: solutions of acetonitrile in distilled water (6–12 vol. %), acidified with HCOOH (10 vol. %).

### 2.5. Spectral Characteristics

Electronic spectra of the anthocyanin' peaks were recorded in DAD cell with a range step of 0.50 nm. Mass spectra were recorded at ESI-mode with column 2.1 × 150 mm Kromasil 100-3.5C18, mobile phase 10 vol. % of HCOOH and 8 vol. % of CH_3_CN in distilled water, 150 mcl/min. Fragmentor voltage of 100 V was applied to get molecular ions and 150 or 200 V to get fragmented ions of corresponding anthocyanidins.

## 3. Results and Discussion

### 3.1. Common Consideration

**Figure 2:**
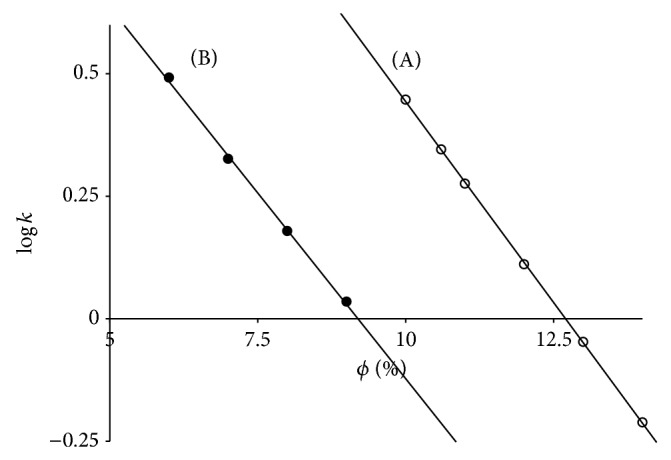
Dependence of Cy3Glu retention upon acetonitrile volume fraction (*ϕ*, %) in System 1 (A) and System 2 (B). Chromatographic column: 4.6 × 250 mm Symmetry C_18_.

The dependence of retention of sample anthocyanin cyanidin-3-glucoside, Cy3Glu (Figure 2), upon volume fraction of CH_3_CN in a mobile phase is rather linear function according to Snyder equation [20] (1) for System 1 (*R* ^2^ = 0.9998) as well as for System 2 (*R* ^2^ = 0.998):

(1) $$\begin{array}{c} \log k_{\phi }\left(i\right)=\log k_{w}\left(i\right)-S\left(i\right)\cdot \phi , \end{array}$$

where *k*_*ϕ*_(*i*) is retention factor of Cy3Glu at a given *ϕ*, volume fraction of CH_3_CN in a mobile phase, and log⁡*k*_*w*_(*i*) and *S*(*i*) are linear approximation constants.

The replacement of 10 vol. % of HCOOH by 0.5 vol. % of phosphoric acid results in pronounced increase of Cy3Glu retention, so that ~3.5 vol. % of CH_3_CN must be added to compensate the increase. Moreover, this means that elution power of HCOOH is three times lower than that of CH_3_CN. Meanwhile for the eluent Systems 1 and 2 parameters *S*(*i*) are 0.162 and 0.150, respectively. The relatively small difference between negative slopes (*S*(*i*)) becomes valuable for the calculated values for the stoichiometric displacement model [20] (2) on the same basis:

(2) $$\begin{array}{c} \log k_{c}\left(i\right)=a-b\cdot \log c\left(\text{C}{\text{H}}_{3}\text{CN}\right), \end{array}$$

where *k*_*c*_(*i*) is retention factor of Cy3Glu at a given* c*(CH_3_CN), molar concentration of CH_3_CN in a mobile phase, and *a* and *b* are linear approximation constants; *b* corresponds to the number of CH_3_CN molecules that are released into a mobile phase during solute sorption.

For System 1 and System 2 parameter *b* is 4.50 and 2.60, respectively. The results indicate a valuable role of HCOOH in sorption-desorption processes and its withdrawal may influence the solute separation selectivity.

### 3.2. Solutes with Different Anthocyanidin Structures and the Same Glycosylation Type

**Figure 3:**
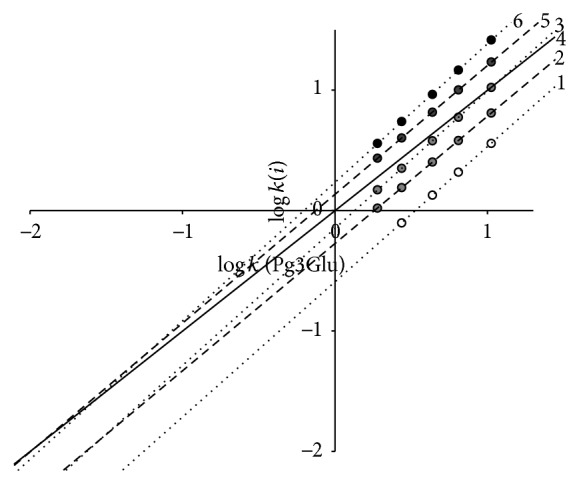
Separation map of 3-glucosides of six common anthocyanins: Dp3Glu (1); Cy3Glu (2); Pg3Glu (3); Pt3Glu (4); Pn3Glu (5); and Mv3Glu (6). System 1. Chromatographic column: 4.6 × 250 mm Symmetry C_18_.

The separation map in the framework of relative retention analysis [17] with pelargonidin-3-glucoside as a reference solute is presented in Figure 3. Each point on the plot has coordinates *x*, logarithm of capacity factor of Pg3Glu and *y*, that for corresponding solute in the same mobile phase. Points for the same solute and different mobile phase compositions settle down on straight lines according to equation of relative retention (3) (Table 1):

(3) $$\begin{array}{c} \log k\left(i\right)=a\cdot \log k\left(\text{Pg}3\text{Glu}\right)+b. \end{array}$$

**Figure 4:**
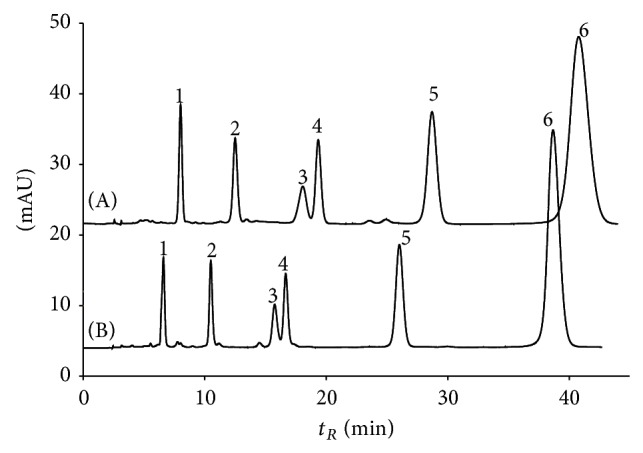
Separation of 3-glucosides of Dp3Glu (1); Cy3Glu (2); Pt3Glu (3); Pg3Glu (4); Pn3Glu (5); and Mv3Glu (6) in mobile phases 0.5 vol. % of H_3_PO_4_ and 9 vol. % of acetonitrile (A) and 10 vol. % of HCOOH and 6 vol. % of acetonitrile (B), 1 mL/min; detection at 515 nm.

**Figure 5:**
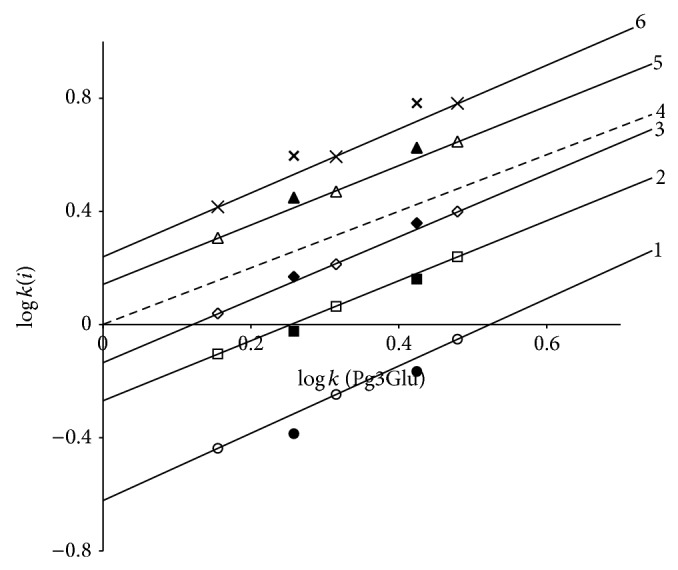
Comparison of separation maps for Dp3Glu (1); Cy3Glu (2); Pg3Glu (3); Pt3Glu (4); Pn3Glu (5); and Mv3Glu (6) in System 1 (empty markers with lines) and in System 2 (corresponding black markers without lines).

Pg3Glu was taken as a reference solute for the simplest ring B structure. Parameters of (3) are the valuable characteristics of corresponding solutes. For example, addition of OH-group into position 3′ of ring B (for a transfer from Pg3Glu to Cy3Glu) leads not only to decrease of retention (and parameter *b*) because of solute hydrophilicity increase but also to increase of parameter *a* as a consequence of additive van der Waals interactions of these O and H atoms with stationary phase atoms. It is easy to see that addition of OH- and CH_3_O-groups to positions 3′ and 5′ of ring B leads to close to additive increase of capacity factor logarithm. This property is true for the same solute retention in System 2 [15]; thus the sequence of anthocyanins' elution on the chromatograms of mixtures of 3-glucosides delphinidin, cyanidin, petunidin, pelargonidin, peonidin, and malvidin for both systems remains unchanged (Figure 4). Meanwhile the exchange of phosphoric acid by HCOOH one results in slight selectivity alterations: some decrease of relative retention for OH-substitutions and an increase of that for OCH_3_-substitutions are evident (Figure 5).

Thus, points for Mv3Glu and Pn3Glu for System 2 settle down above the lines for the same anthocyanins for System 1, while the opposite case is found for relative retention of Dp3Glu and Cy3Glu. Accordingly, System 2 has somewhat higher selectivity for separation of substances with different flavylium ions hydrophilicity.

The lines of relative retention approximated to the region of zero points [21] (left down corner of the plot on Figure 3) are differentiated according to the number of OH-groups in ring B of anthocyanin structure. Moreover, according to our observation the place of OH-addition has no meaning—the particular anthocyanins of* Alstroemeria* flowers [22] with OH-groups at carbon atoms number 6 have retention close to that of isomers with OH-groups in ring B. This is in a full agreement with the property of reversed-phases chromatography having a low selectivity for isomers separation. Accordingly, the position of lines of relative retention (3) in the region of zero points may be utilised for tentative chromatographic estimation of the number of OH-groups in solute molecules.

### 3.3. Solutes with the Same Anthocyanidin Structure and Different Glycosylation Type

**Figure 6:**
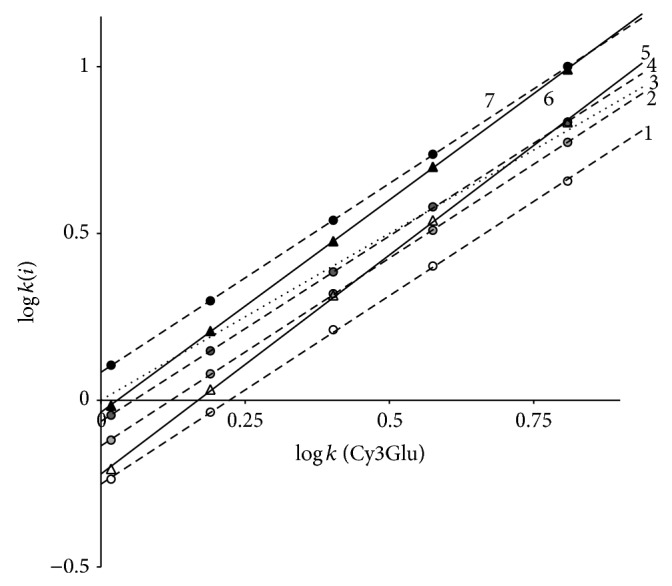
Separation map of cyanidin-3-glycosides: Cy3Sopho (1); Cy3AGlu (2); Cy3Glu (3); Cy3Sam (4); Cy3GRut (5); Cy3XRut (6); and Cy3Rut (7) in System 1. Chromatographic column: 4.6 × 250 mm Symmetry C_18_.

The separation map of relative retention of some cyanidin-3-glycosides with Cy3Glu as a reference solute in System 1 is presented in Figure 6. It becomes evident that not only the absolute retention but also relative retention of different cyanidin-3-glycosides depends not only upon solute structure, but also upon mobile phase composition; coelution of some solute pairs including the reversal of the elution order may occur after alteration of component concentrations in the mobile phase of the same eluent system and stationary phase.

By the way the drawing of the resolution map may escape mistakes connected with estimation of number of solutes and choice of appropriate mobile phase composition for complex mixtures analysis by HPLC methods.

In the case of System 1 parameter *a* of (3) may be utilised for preliminary estimation of complexity of glycoside structure in position 3 of anthocyanidin backbone: *a* has a value in the region 1.000 ± 0.020 for monoglycosides (Cy3Gala and Cy3Ara), 1.122 ± 0.011 for diglycosides (Cy3Sopho, Cy3Sam, Cy3AGlu, and Cy3Rut), and 1.300 ± 0.030 for triglycosides (Cy3GRut, Cy3XRut) (Table 2). The values are close to that reported for retention of cyanidin-3-glycosides in RP HPLC in solvent System 2 [17, 23] proving the property to be a common regularity of the solute chromatographic behaviour at least for the systems under investigation.

**Figure 7:**
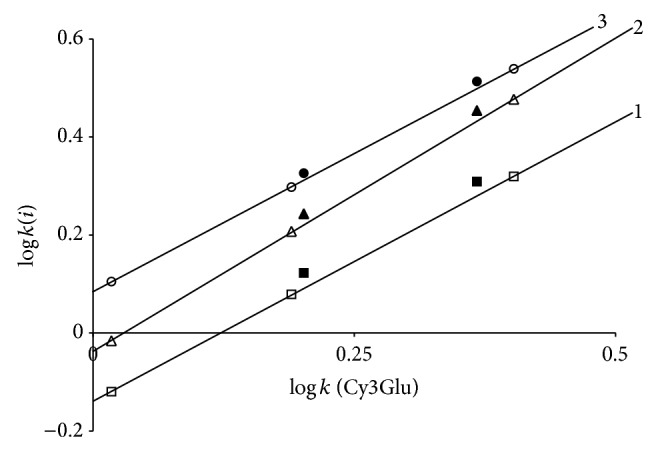
Comparison of separation maps for Cy3AGlu (1); Cy3XRut (2); and Cy3Rut (3) in System 1 (empty markers with lines) and in System 2 (corresponding black markers without lines).

Finely, the exchange of HCOOH by phosphoric acid also leads to slight decrease of relative retention of di- and trisaccharides, Figure 7, but no significant alterations of solutes separation selectivity were found.

## 4. Conclusions

The exchange of HCOOH (System 1) for phosphoric acid (System 2) leads to substantial increase of anthocyanins retention in mobile phases, acidified mixtures of water and acetonitrile.

Selectivity of resolution of the same glycosides of six common anthocyanidins is only slightly greater in System 2, by the way, though the sequence of elution remains the same for reasonable solutes retention times.

Analysis of anthocyanins relative retentions on the separation map (in the region of zero points) may be explored for estimation of the number of OH-groups in anthocyanidin backbone.

Selectivity of resolution of the different glycosides of the same anthocyanidin (an example of the most common natural aglicone, cyanidin) is also close to that for System 1.

But the advantage of relative retention analysis is a sensitivity to structure of carbohydrate radicals; parameter *b* for mono-, di-, and trisaccharides is differing enough permitting determination of complexity of glycosyl radical in the 3 positions of anthocyanidin without utilization of MS detection. Moreover, parameter *a* is highly sensitive to sugar isomers structures.

Thus, eluent systems under investigation have close properties, though System 2 seems to be somewhat more efficient.

## Figures and Tables

**Table 1:** Chromatographic characteristics of 3 glucosides of six common anthocyanidins.

*N*	Solute	Parameters of (3)			*λ*_max⁡_^a^, nm	*m*/*z*
		*a*	*b*	*R* ^2^		
1	Dp3Glu	1.123 ± 0.010	−0.618	0.99995	523.0	465.1; 287.0
2	Cy3Glu	1.057 ± 0.008	−0.273	0.99998	515.0	449.1; 287.0
3	Pt3Glu	1.139 ± 0.011	−0.146	0.99997	524.0	479.2; 317.0
4	Pg3Glu	1	0	—	500.5	433.2; 271.0
5	Pn3Glu	1.070 ± 0.010	0.136	0.99997	515.0	463.1; 301.0
6	Mv3Glu	1.150 ± 0.010	0.236	0.9999	525.0	493.1; 331.0

^a^Mobile phase: 10 vol.% CH_3_CN and 0.5 vol.% H_3_PO_4_.

**Table 2:** Parameters of relative retention (3) of some cyanidin-3-glycosides.

Solutes	System 1			System 2 [23]		*m*/*z*
	*a*	*b*	*R* ^2^	*a*	*b*	
Cyanidin-3-monoglycosides						
Galactoside, Cy3Gala	0.983 ± 0.012	−0.122	0.9998	0.976	−0.135	449.1; 287.0
Glucoside, Cy3Glu	1	0	—	1	0	449.1; 287.0
Arabinoside, Cy3Ara	0.941 ± 0.011	0.140	0.9998	0.932	0.141	419.1; 287.0

Cyanidin-3-diglycosides						
Glucosylglucoside, Cy3Sopho	1.130 ± 0.012	−0.251	0,9997	—	—	611.1; 287.0
Xylosylglucoside, Cy3Sam	1.112 ± 0.008	−0.063	0,99997	1.148	−0.066	581.1; 287.0
Rhamnosylglucoside, Cy3Rut	1.133 ± 0.007	0.084	0,99999	1.131	0.099	595.2; 287.0
Arabinosylglucoside, Cy3AGlu	1.126 ± 0.010	−0,137	0,99995	1.138	−0.110	581.1; 287.0

Cyanidin-3-triglycosides						
Glucosylrutinoside, Cy3GRut	1.313 ± 0.013	−0.221	0.9997	1.308	−0.235	757.2; 287.0
Xylosylrutinoside, Cy3XRut	1.273 ± 0.008	−0.036	0.99996	1.285	−0.057	727.2; 287.0
